# Unraveling the Saline–Alkali–Tolerance Mystery of *Leymus chinensis* Nongjing–4: Insights from Integrated Transcriptome and Metabolome Analysis

**DOI:** 10.3390/plants14243852

**Published:** 2025-12-17

**Authors:** Jianli Wang, Mingyu Wang, Zijian Zhang, Jinxia Li, Qiuping Shen, Yuanhao Zhang, Dongmei Zhang, Linlin Mou, Xu Zhuang, Wenhui Wang, Zhaohui Li, Long Han, Zhongbao Shen, Lixin Li

**Affiliations:** 1 Institute of Forage and Grassland Sciences, Heilongjiang Academy of Agricultural Sciences, Harbin 150086, China; jianli@haas.cn (J.W.); budaoweng@haas.cn (L.M.); yangyangcaocao@haas.cn (X.Z.); wangwenhui@163.com (W.W.); lzhh010319@163.com (Z.L.); 2 Key Laboratory of Saline–Alkali Vegetation Ecology Restoration, Ministry of Education, College of Life Sciences, Northeast Forestry University, Harbin 150040, China; wmy19970825@163.com (M.W.); zhangzijian200107@163.com (Z.Z.); lijinxia@nefu.edu.cn (J.L.); shenqiuping2021@163.com (Q.S.); zhangyuanhao1011@163.com (Y.Z.); zhangdongmei@haas.cn (D.Z.); hanlharry@163.com (L.H.); 3 College of Landscape Architecture, Northeast Forestry University, Harbin 150040, China

**Keywords:** flavonoid biosynthesis, stress–severity–dependent response, integrated omics, resistant forage cultivar

## Abstract

Soil salinization–alkalization is a critical abiotic constraint on global agriculture, threatening agroecosystem sustainability. *Leymus chinensis*, a high–quality perennial forage with strong stress resilience, is an ideal model for studying saline–alkali tolerance in graminaceous crops. We integrated physiological, transcriptomic, and metabolomic profiling to dissect its responses under moderate vs. severe carbonate stress, mimicking natural saline–alkali soils rather than single salt stress treatments. Multi–omics analysis revealed drastic reprogramming of energy metabolism, carbohydrate homeostasis, water transport, and secondary metabolism. Our novel finding reveals that *L. chinensis* uses stress–severity–dependent mechanisms, with flavonoid biosynthesis as a central “regulatory hub”: moderate saline–alkali stress acts as a stimulus for “Adaptive Activation” (energy + antioxidants), promoting growth, while severe stress exceeds tolerance thresholds, causing “systemic imbalance” (oxidative damage + metabolic disruption) and growth retardation. Via WGCNA and metabolome–transcriptome modeling, 22 transcription factors linked to key flavonoid metabolites were identified, functioning as molecular switches for stress tolerance. Our integrated approach provides novel insights into *L. chinensis*’ tolerance networks, and the flavonoid biosynthesis pathways and regulatory genes offer targets for precision molecular breeding to enhance forage stress resistance and mitigate yield losses from salinization–alkalization.

## 1. Introduction

Saline–alkali stress poses a significant threat to agricultural production, as the expansion of land with high Na^+^ concentration and pH reduces arable area and limits crop yields [1]. Therefore, improving saline–alkali land and breeding tolerant crops have become two main strategies to address these constraints by expanding arable land and boosting productivity [2,3]. In China, the total area of saline–alkali land is approximately 9.91 × 10^7^ hectares [4]. Notably, the Songnen Plain in Northeast China is dominated by carbonate–rich saline–alkali soils, earning it the name “soda saline–alkali land” [5].

Alkaline salt stress is more complex than salt stress alone. In addition to ionic toxicity and osmotic stress caused by salt stress, the presence of carbonate and bicarbonate ions increases soil pH, leading to pH stress and more severe oxidative damage [6,7]. Saline–alkali stress impairs the integrity of the cell membrane, reduces photosynthetic rate, disrupts ROS (reactive oxygen species) homeostasis, and causes other serious damage. Furthermore, the high pH value of the soil converts many nutrients into insoluble compounds, which cannot be absorbed by plants, leading to an imbalance of nutrient elements [8]. Saline–alkali stress inhibits various growth stages of plants, ultimately reducing plant biomass and yield. For example, in *L. barbarum*, the germination rate decreases from 90% to approximately 60% under 300 mM NaHCO_3_:Na_2_CO_3_ (1:1) and to less than 20% at 600 mM [9]. Treatment with 120 mM NaHCO_3_ significantly reduces the biomass, net photosynthetic rate (P_n_), stomatal conductance (g_s_), and transpiration rate (T_r_) of *Ricinus communis* L. [10]. Furthermore, saline–alkali stress disrupts plant metabolism, leading to significant changes in the contents of organic acids, amino acids, sugars, and flavonoids in quinoa [11] and cotton [12]. These studies indicate that the toxicity of saline–alkali stress to plants is multifaceted. Thus, exploring plant response mechanisms to saline–alkali stress, including related physiological and metabolic processes, is crucial for providing an important theoretical basis for breeding tolerant crops and germplasm innovation.

Long–term evolution has enabled plants to develop complex environmental adaptation mechanisms to ensure survival and growth under saline–alkali stress, including osmotic adjustment, exclusion or sequestration of excess Na^+^, and regulation of ROS homeostasis [13,14,15]. To eliminate excessive ROS, plants have evolved two major pathways: enzymatic and non–enzymatic antioxidant systems [16]. The enzymatic pathway primarily involves superoxide dismutase (SOD), peroxidase (POD), catalase (CAT), and ascorbate peroxidase (APX), which collectively convert ROS into harmless molecules [17,18,19,20]. The non–enzymatic pathways involve antioxidants such as ascorbic acid, carotenoids, flavonoids, and phenolic compounds, which directly scavenge free radicals or interrupt free radical chain reactions, indirectly protecting membrane integrity and metabolic stability [21,22,23]. Flavonoids are a class of polyphenolic compounds widely present in plants, with a C6–C3–C6 skeleton synthesized via the phenylpropanoid pathway, and play a crucial role in antioxidant defense. Flavonoids are divided into several subclasses, including flavones, isoflavones, flavanones, flavanols, chalcones, and anthocyanins [24]. These compounds possess strong antioxidant properties and regulate plant physiological processes and environmental interaction. Under saline–alkali stress, flavonoids play a vital role in enhancing plant stress resistance [25,26]. For example, flavonoid biosynthesis in the alfalfa resistant cultivar NQ–1 grown in soda saline–alkali soil plays an important role in stress adaptation [27]. Quinoa is a saline–alkali–tolerant species, and Wang et al. systematically summarized the flavonoids, synthases, and transcription factors of the MBW complex that function in the Flavonoid biosynthesis pathway of quinoa in response to saline–alkali stress [28]. Isoliquiritigenin, a chalcone, exhibits antioxidant and cytoprotective effects that alleviate ROS–induced oxidative damage to plant cells under saline–alkali stress [29]. Pelargonidin and cyanidin, anthocyanins with antioxidant properties, may help maintain normal plant photosynthesis under saline–alkali stress by regulating plant pigment metabolism [30].

*Leymus chinensis* (2n = 4x = 28, NsNsXmXm), a perennial herbaceous species, is widely distributed across the Eurasian steppe. Endowed with high nutritional value, strong palatability, and a remarkable tolerance to environmental stresses, including saline–alkali, drought, and freezing temperatures [31,32,33], *L. chinensis* serves as an important forage crop [34]. As a dominant species, it fulfills crucial ecological and agronomic roles, supporting livestock production and maintaining steppe ecosystem stability. For drought tolerance, its deep rhizome system and efficient water–use efficiency are critical adaptive traits, though the molecular mechanisms require further investigation. Notably, it thrives in aeolian soils with pH > 7.0, where fertilization can boost yield by 74.7% compared to unfertilized controls, highlighting its adaptability to marginal lands [35]. *L. chinensis* exhibits severity–dependent physiological and metabolic responses to saline–alkaline stress. Under neutral salt stress, low–concentration treatment (100 mM NaCl) triggers significant accumulation of soluble sugars, free amino acids, and betaine, in leaves, whereas high–concentration salt stress (400 mM) predominantly induces organic acid synthesis [36,37]. Notably, alkaline stress imposes more severe deleterious effects than neutral salt stress: exposure to 25 mM Na_2_CO_3_ simultaneously elevates the levels of soluble sugars, amino acids, betaine, and organic acids, while mixed alkaline salts (Na_2_CO_3_:NaHCO_3_ = 1:1, 45 mM Na^+^) markedly inhibit seedling growth and photosynthetic activity, leading to ionic imbalance and carbon deficiency [38]. Intraspecific variations in stress tolerance are evident among different ecotypes. In moderately saline–alkaline soils (MS, pH 8.5–9.5), both the gray–green (GG) and yellow–green (YG) ecotypes of *L. chinensis* maintain leaf area, tiller biomass, net photosynthetic rate (Pn), and potassium content at levels comparable to or even higher than those under lightly saline–alkaline conditions (LS, pH 7.1–8.5). However, under severe saline–alkaline stress (SS, pH > 9.5), the GG ecotypes outperform the YG ecotype, with higher Pn and chlorophyll concentrations coupled with lower Na^+^ accumulation, thereby sustaining growth and physiological functionality more effectively [39]. With the release of high–quality genomes of *L. chinensis* [40], molecular–level research on it is receiving increasing attention. Integrative transcriptomic and metabolomic analyses have revealed that pathways such as photorespiration, GSH/GSSH redox cycling, and ABA signaling are activated under stress, with key metabolites (serine, glycolate) and enzymes (glycolate oxidase) modulating stress responses [41]. Several key structural genes orchestrating these adaptive responses have been cloned and functionally characterized, including betaine aldehyde dehydrogenase (*LcBADH*) [42], glyceraldehyde–3–phosphate dehydrogenase (*LcGAPC*) [43], chitinase 2 (*LcCHI2*) [44], oil body–associated protein (*LcOBAP2B*) [45], and metallothionein–3 (*LcMT3*) [46]. These validated genes represent promising candidates for molecular breeding programs aimed at enhancing saline–alkaline tolerance in *L. chinensis*. Collectively, current research has primarily focused on single NaCl or Na_2_CO_3_ stress, while the synergistic effects of alkaline salts (Na_2_CO_3_/NaHCO_3_) and high pH remain limited.

In order to reveal the stress adaptation mechanism of saline–tolerant *Leymus chinensis* variety NQ–4, we treated *L. chinensis* with moderate and high concentrations of carbonate solutions and then conducted physiological, transcriptomic, and metabolomic analyses. The results showed that *L. chinensis* had different response mechanisms to moderate and severe saline–alkali stress. Our findings provide a theoretical basis for future research on the salt alkali stress response of *Leymus chinensis* and support genetic improvement of this species in saline alkali cultivation.

## 2. Results

### 2.1. Growth Performance and Physiological Activities of Leymus chinensis Under Saline–Alkali Stress

**Figure 1:**
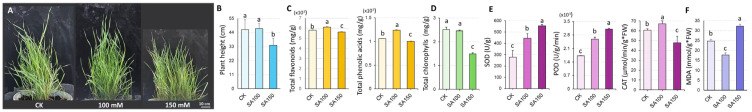
Growth performance and physiological activities of *Leymus chinensis* under saline–alkali stress. (**A**) Growth performance of one–year–old *L. chinensis* after 100 mM and 150 mM mixed carbonate solution (Na_2_CO_3_:NaHCO_3_ = 1:9) treatment for 14 days. CK, control group. (**B**–**F**) Statistics for plant height (*n* ≥ 30) (**B**); contents of total flavonoids and phenolic acids in the leaves (*n* ≥ 3) (**C**); chlorophyll contents (*n* ≥ 3) (**D**); activities of antioxidant enzymes (*n* ≥ 3) (**E**); and MDA content (*n* ≥ 3) (**F**) Data are means ± SE. The different letters indicate a significant difference, *p* < 0.05.

To elucidate the saline–alkali (SA) stress response mechanism of *L. chinensis*, Nongjing–4 (NQ–4), a resistant cultivar registered as a crop cultivar in Heilongjiang Province (Registration No. 2008008), was selected for comprehensive analysis. One–year–old plants were treated with 100 mM and 150 mM mixed carbonate solutions (Na_2_CO_3_:NaHCO_3_ = 1:9, ~pH9.3) for 14 days, and their growth performance was observed. The plant height showed no significant change under the moderate carbonate concentration (100 mM, SA100), whereas a significant reduction was observed under the high carbonate concentration (150 mM, SA150) compared to the control group (CK) (Figure 1A,B). The contents of total flavonoids and phenolics in leaves significantly increased in the SA100 group but decreased significantly in the SA150 group (Figure 1C). While, the total chlorophyll-contents significantly decreased in the SA150 group, but no significance in the SA100 group (Figure 1D). These results demonstrated that severe saline–alkali stress not only inhibits the growth and development of NQ–4 but also significantly affects its metabolism, whereas moderate saline–alkali stress promotes NQ–4 growth and development to some extent.

Since saline–alkali stress induces a burst of ROS, which causes oxidative damage to plant cells [22], the activities of antioxidant enzymes and malondialdehyde (MDA), an oxidative damage marker, in leaves were detected. The activities of POD and SOD significantly increased in both the SA100 and SA150 groups. However, CAT activity significantly increased in the SA100 group but decreased in the SA150 group, and the MDA content significantly decreased in the SA100 group but increased in the SA150 group (Figure 1E,F), indicating that saline–alkali stress resulted in oxidative damage and activated the antioxidant system. These results indicated that under saline–alkali stress, ROS homeostasis in NQ–4 was disrupted, leading to activation of the antioxidant machinery to acquire resistance. Specifically, under moderate saline–alkali stress, the resistance of NQ–4 was enhanced, and its growth was improved. However, under severe saline–alkali stress, the damage caused by stress was much greater than the resistance acquired by NQ–4, resulting in significant growth inhibition.

### 2.2. Transcriptomic Analysis of Leymus chinensis Under Saline–Alkali Stress

**Figure 2:**
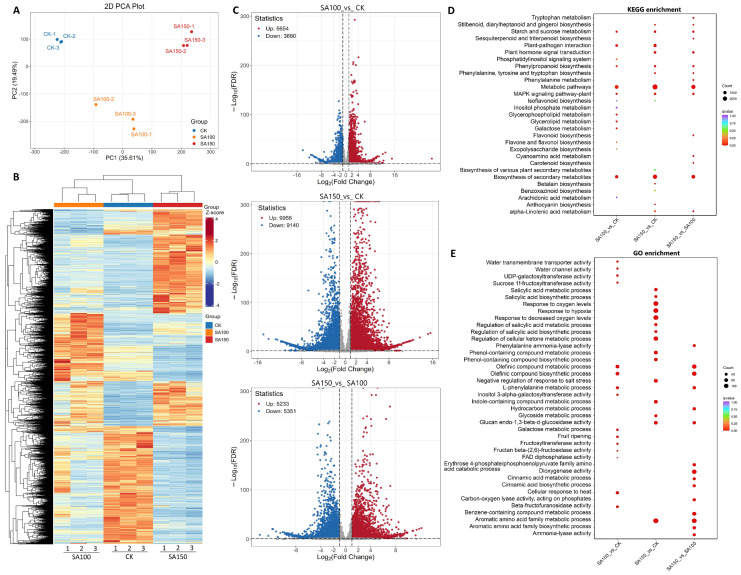
Transcriptomic analysis of leaves of *Leymus chinensis* under saline–alkali stress. (**A**). Principal component analysis (PCA) diagram. X–axis and Y–axis indicate the first and second principal components (PC1 and PC2), respectively. Scores of PC1 and PC2 show cohesion within groups and separation between the CK, SA100, and SA150 groups. (**B**). Heatmap of CK, SA100, and SA150 groups. (**C**). Volcano plots of SA100_vs_CK, SA150_vs_CK, and SA150_vs_SA100 groups. VIP > 1, *p* value < 0.05. Red dots, upregulated genes; blue dots, downregulated genes; gray dots, genes with no significance. (**D**). KEGG enrichment analysis. (**E**). GO enrichment analysis. The *p*-value is presented in a color scale, and the size of the dots represents the DEG number mapped in each pathway.

To elucidate the gene response of NQ–4 to saline–alkali stress, we analyzed the transcriptome of leaves grown under 100 mM and 150 mM carbonate treatments. Principal component analysis (PCA) revealed significant differences among the CK, SA100, and SA150 groups (Figure 2A). The total ion flow (TIC) diagram shows that the total ion current curve of metabolite detection had high overlap; i.e., the retention time and peak intensity were consistent, indicating that the signal stability was good when mass spectrometry was used to detect the same sample at different times. The high stability of the instrument provides an important guarantee for the repeatability and reliability of the data (Figure S1). The heatmap shows distinct differences in transcript profiles among CK, SA100, and SA150 (Figure 2B). In the 100 mM carbonate treatment and CK comparison group (SA100_vs_CK), 9334 differentially expressed genes (DEGs) were identified, including 3680 downregulated and 5654 upregulated genes. For the SA150_vs_CK group, 19,096 DEGs were identified, with 9140 downregulated and 9956 upregulated. For the SA150_vs_SA100 group, 10,584 DEGs were identified, with 5351 downregulated and 5233 upregulated (Figure 2C). These results indicate that a higher concentration of carbonate solution exerted a more profound impact on the gene transcriptions in NQ–4.

To further elucidate the molecular mechanisms underlying NQ–4 responses to saline–alkali stress, KEGG and Gene Ontology (GO) enrichment analyses were conducted. KEGG analysis indicated that across the three comparisons, DEGs were primarily enriched in the pathways such as Stilbenoid, diarylheptanoid, and gingerol biosynthesis, Starch and sucrose metabolism, Phenylpropanoid biosynthesis, MAPK signaling pathway–plant, Plant hormone signal transduction, and alpha–linolenic acid metabolism, in addition to Metabolic pathways and Biosynthesis of secondary metabolites pathways (Figure 2D). GO analysis revealed that for the SA100_vs_CK comparison group, DEGs were enriched in terms including water transmembrane transporter activity, water channel activity, UDP–galactosyltransferase activity, and sucrose 1f–fructosyltransferase activity. For the SA150_vs_CK comparison group, DEGs were enriched in stress–responsive processes such as salicylic acid metabolic process, salicylic acid biosynthetic process, response to oxygen levels, response to hypoxia, and regulation of salicylic acid metabolic process (Figure 2E). Notably, both KEGG and GO enrichment were predominantly associated with sugar and energy component biosynthesis and metabolism, suggesting that energy production and conversion in NQ–4 are significantly affected under saline–alkali stress.

### 2.3. Widely Targeted Metabolomics Analysis of Differentially Accumulated Metabolites

**Figure 3:**
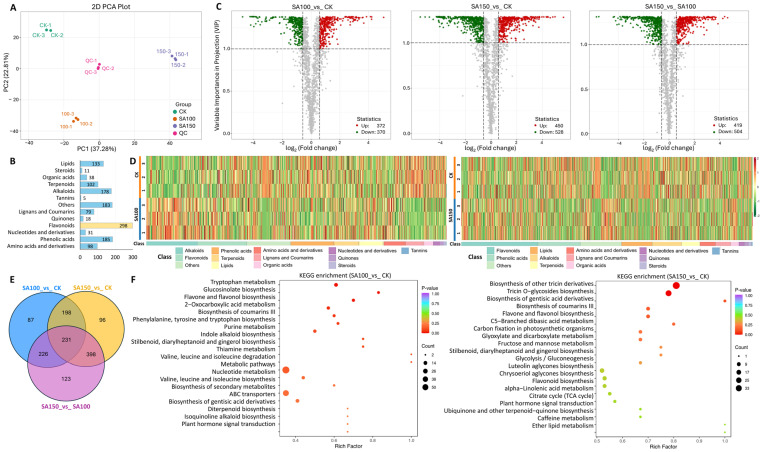
Widely targeted metabolomics analysis of differentially accumulated metabolites. (**A**) PCA plots of CK, SA100, and SA150. (**B**) Enrichment of the DAMs in the indicated metabolite categories. (**C**) Volcano plots of SA100_vs_CK, SA150_vs_CK, and SA150_vs_SA100 groups. Red dots, upregulated DAMs; green dots, downregulated DAMs; gray dots, no significance. (**D**) Heatmap of the DAMs in the indicated categories. (**E**) Venn diagram visualizing the distribution of DAMs in SA100_vs_CK, SA150_vs_CK, and SA150_vs_SA100 groups. (**F**) KEGG enrichment analysis of SA100_vs_CK and SA150_vs_CK groups.

To elucidate the patterns of metabolite changes in *L. chinensis* leaves under saline–alkali stress, metabolic profiles were determined using ultraperformance liquid chromatography–electrosprayionization–tandem mass spectrometry (UPLC–ESI–MS/MS)–based metabolomics. Principal component analysis (PCA) revealed significant differences in metabolite profiles among the CK, SA100 mM, and SA150 mM groups (Figure 3A). A total of 1360 metabolites were detected by total ion chromatography (TIC), including 298 flavonoids, 185 phenolic acids, 178 alkaloids, and 133 lipids (Figure 3B). In the SA100_vs_CK group, 372 differently accumulated metabolites (DAMs) were upregulated, while 370 were downregulated. For the SA150_vs_CK group, 450 DAMs were upregulated and 528 downregulated; for the SA150_vs_SA100 group, there were 419 DAMs upregulated and 504 downregulated (Figure 3C). heatmap_class_overall showed that in the SA100_vs_CK group, alkaloids, flavonoids, phenolic acids, terpenoids, and lipids contained the highest number of DAMs. In contrast, the SA150_vs_CK group showed the highest DAM counts in the order of flavonoids, Phenolic acids, lipids, alkaloids, and terpenoids (Figure 3D), indicating distinct response mechanisms to moderate versus severe saline–alkali stress. The Venn diagram demonstrates 231 common DAMs across the three groups, with 87, 96, and 123 unique DAMs exclusive to the SA100_vs_CK, SA150_vs_CK, and SA150_vs_SA100 groups, respectively (Figure 3E). KEGG enrichment analysis indicated that for the SA100_vs_CK group, DAMs were mainly enriched in pathways including tryptophan metabolism, glucosinolate biosynthesis, flavone and flavonol biosynthesis, 2–oxocarboxylic acid metabolism, biosynthesis of coumarins III, etc. For the SA150_vs_CK group, DAMs were primarily enriched in biosynthesis of other tricin derivatives, tricin O–glycosides biosynthesis, biosynthesis of gentisic acid derivatives, biosynthesis of coumarin III, and flavone and flavonol biosynthesis. These results collectively suggest that flavonoid biosynthesis and metabolism play a crucial role in response to saline–alkali stress in NQ–4.

### 2.4. Combined Transcriptome and Metabolome Analysis of Saline–Alkali Stress Response in NQ–4 Leaves

**Figure 4:**
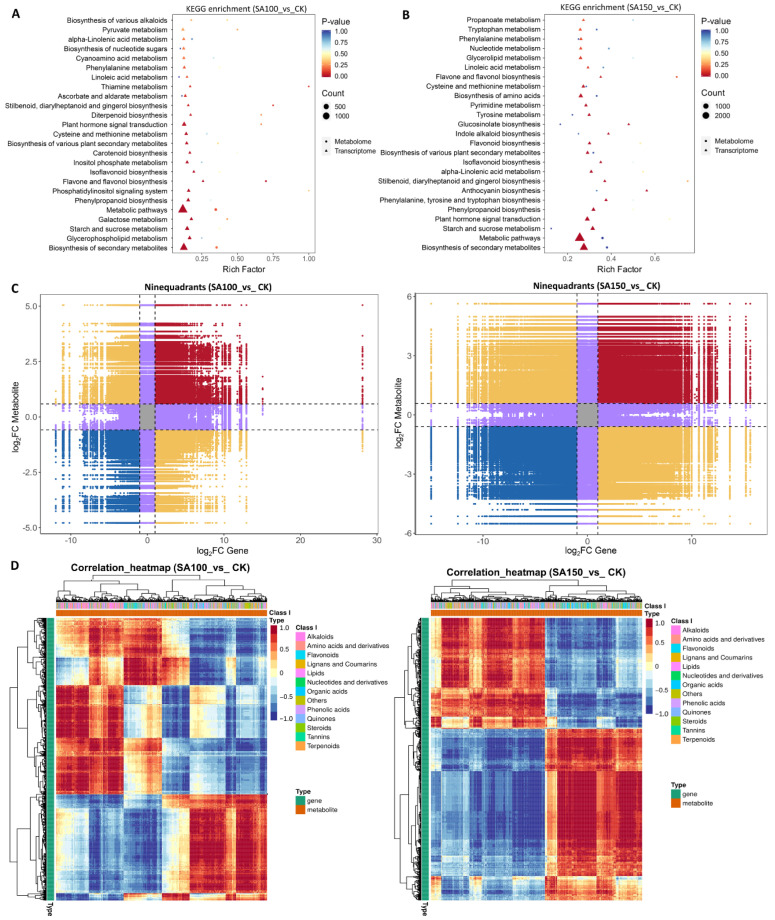
Combined transcriptome and metabolome analysis of saline–alkali stress response in NQ–4 leaves. (**A**,**B**) KEGG enrichment of DEGs and DAMs in SA100_vs_CK (**A**) and SA150_vs_CK (**B**) groups. (**C**) Nine–quadrant diagrams visually display the correlation patterns between gene expression and metabolite accumulation. (**D**) Heatmap of gene–metabolite correlation clustering.

Integrated omics analysis enables a comprehensive understanding of plant responses to saline–alkali stress. KEGG pathway enrichment from both transcriptomic and metabolomic data was visualized using bubble diagrams. Under the 100 mM carbonate treatment, DEGs and DAMs were enriched in pathways including glycerophospholipid metabolism, galactose metabolism, starch and sucrose metabolism, phenylpropanoid biosynthesis, phosphatidylinositol signaling system, and flavone and flavonol biosynthesis (Figure 4A). On the other hand, under 150 mM carbonate treatment, DEGs and DAMs were enriched in starch and sucrose metabolism, plant hormone signal transduction; phenylpropanoid biosynthesis; phenylalanine, tyrosine, and tryptophan biosynthesis; anthocyanin biosynthesis; Stilbenoid, diarylheptanoid, and gingerol biosynthesis; alpha–linolenic acid metabolism; isoflavonoid biosynthesis; and flavonoid biosynthesis (Figure 4B). These findings indicate that NQ–4 adopts distinct mechanisms to cope with moderate and severe saline–alkali stress.

Notably, among the top 20 enriched KEGG pathways, isoflavonoid biosynthesis, flavone and flavonol biosynthesis, and phenylpropanoid biosynthesis exhibited significant alterations, suggesting that flavonoid biosynthesis plays a critical role in response to saline–alkali stress in NQ–4. A nine–quadrant diagram summarizes the correlations between metabolites and genes, illustrating their diverse relationships. Quadrants 1–9, arranged from left to right and top to bottom, are separated by black dotted lines (Figure 4C). Quadrant 5 (center gray area) contains genes and metabolites with no differential expression/accumulation. Quadrants 3 and 7 (red and blue) show consistent differential expression/accumulation patterns between genes and metabolites, indicating a positive correlation and suggesting that metabolite biosynthesis is positively regulated by these genes. Quadrants 1 and 9 (yellow) display opposite patterns, implying negative regulation of metabolite biosynthesis by genes. Quadrants 2, 4, 6, and 8 (purple) exhibit unsynchronized changes, with one gene or metabolite showing differences, while the other remains unchanged.

Additionally, a correlation clustering heatmap was generated to visualize gene–metabolite correlations, where red indicates positive correlations, and blue indicates negative correlations (Figure 4D). Both the nine–quadrant diagram and correlation heatmaps revealed distinct correlation patterns between the SA100_vs_CK and SA150_vs_CK groups, confirming that NQ–4 employs different response mechanisms to moderate and severe saline–alkali stress.

### 2.5. Change Patterns of Flavonoids Under Saline–Alkali Stress

**Figure 5:**
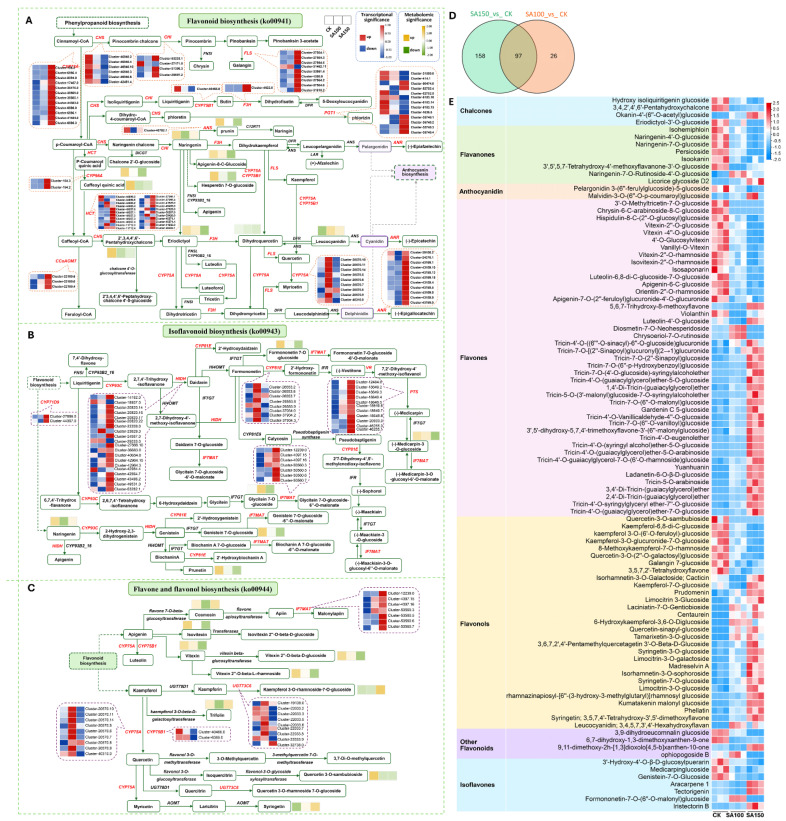
Change patterns of flavonoids under saline–alkali stress. (**A**–**C**) Statistics of expression levels of DEGs and accumulation levels of DAMs in flavonoid biosynthesis, isoflavonoid biosynthesis, and flavone and flavonol biosynthesis pathways. The red names highlight the DEGs, and the rectangles represent the metabolites. The significances of the flavonoids are indicated in the triple blocks. The solid lines with arrows indicate directions of the processes, and the dotted lines indicate connections to other metabolism pathways. (**D**) Venn diagram visualizing the distribution of flavonoids in SA100_vs_CK and SA150_vs_CK groups. (**E**) The variation in the accumulation of 97 flavonoids shared between the CK, SA100, and SA150 groups.

A total of 298 flavonoids detected in *L. chinensis* via widely targeted metabolomics can be classified into ten subclasses, namely chalcones, aurones, flavanones, flavanonols, anthocyanidins, flavones, flavonols, flavanols, isoflavones, and other flavonoids (Table S1). The accumulation levels of DAMs and expression levels of DEGs enriched in the flavonoid biosynthesis (ko00941), isoflavonoid biosynthesis (ko00943), and flavone and flavonol biosynthesis (ko00944) pathways are summarized in Figure 5A–C. Among the differentially accumulated flavonoids, 97 were commonly identified in both the SA100_vs_CK and SA150_vs_CK groups, while 26 and 158 were specific to the SA100_vs_CK and SA150_vs_CK groups, respectively (Figure 5D). Statistical analysis of the accumulation levels of these 97 common flavonoids revealed significant gradient effects: most metabolites exhibited more pronounced changes under the 150 mM carbonate treatment compared to the 100 mM treatment (Figure 5E). These findings provide compelling evidence supporting the hypothesis that flavonoids are involved in the response to saline–alkali stress. Furthermore, they provide potential targets for molecular design breeding, which could enhance plant saline–alkali stress tolerance in the future by modifying these key metabolic pathways.

### 2.6. Identification of Candidate Genes Encoding Synthetic Enzyme and Transcription Factor Genes Involved in Flavonoid Biosynthesis Under Saline–Alkali Stress

**Figure 6:**
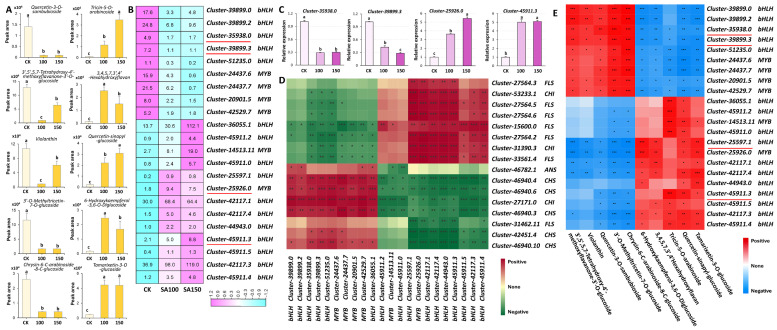
Identification of candidate biosynthetic enzyme and transcription factor genes involved in flavonoid biosynthesis. (**A**) Statistics of the relative contents of the five most significantly upregulated and five most downregulated flavonoids. (**B**) Heatmap of transcriptional levels of the 22 TF genes regulating flavonoid synthesis under saline–alkali stress. Red lines, the TFs validated by RT–qPCR. (**C**) Statistics of the relative expression levels of the indicated genes validated by RT–qPCR. *UBQ10* was used as an endogenous control. Three independent experiments per sample and three technical replicates per experiment. Data are means ± SE. The letters above the error bars indicate significance at *p* < 0.05. (**D**) Correlations between the 22 TF genes and the biosynthetic enzyme genes. (**E**) Correlations between the 22 TF genes and the flavonoids in (A). * *p* < 0.05; ** *p* < 0.01; *** *p* < 0.001.

To further investigate the impact of saline–alkali stress on flavonoids, we conducted comprehensive statistical analyses of the changes in flavonoid content, focusing on ten flavonoids. Among them, five flavonoids showed the most significant increase, and five flavonoids showed the most significant decrease under stress treatment. The most upregulated flavonoids were 6–hydroxykaempferol–3,6–O–diglucoside, quercetin–sinapyl–glucoside, tamarixetin–3–O–glucoside (tamarixin), leucocyanidin (3,4,5,7,3′,4′–hexahydroxyflavan), and tricin–5–O–arabinoside, and the most downregulated flavonoids were 3′–O–methyltricetin–7–O–glucoside, chrysin–6–C–arabinoside–8–C–glucoside, quercetin–3–O–sambubioside, 3′,5′,5,7–tetrahydroxy–4′–methoxyflavanone–3′–O–glucoside, and violanthin (Figure 6A). During flavonoid biosynthesis, the MBW complex, composed of MYB, bHLH, and WD40 proteins, plays a pivotal regulatory role. To elucidate the regulatory functions of the MYB and bHLH transcription factors (TFs) in flavonoid biosynthesis in *L. chinensis* under saline–alkali stress, 22 candidate TF genes were identified via correlation analysis, including 16 bHLH family members and 6 MYB family members. Under treatment with 100 mM and 150 mM carbonate solutions, 9 of these TF genes were downregulated, while 13 were upregulated (Figure 6B). RT–qPCR validation of selected TF genes confirmed expression trends consistent with transcriptome data (Figure 6C). Furthermore, we selected flavonoid biosynthetic enzyme genes (*CHS*, *CHI*, *FLS*, and *ANS*), as indicated in Figure 5, and analyzed their correlation with these 22 identified TFs. The results revealed a strong correlation between the expression profiles of these TFs and the flavonoid biosynthetic enzyme genes (Figure 6D). Specifically, two *bHLH* TF genes, *cluster–35938.0* and *cluster–39899.3*, exhibited strong positive correlations with the five most downregulated flavonoids, while showing negative correlations with the five most upregulated ones. In contrast, *cluster–25597.1* (a *bHLH* TF gene) and *cluster–25926.0* (a *MYB* TF gene) displayed strong negative correlations with the five most downregulated flavonoids and positive correlations with the five most upregulated ones, except for tricin–5–O–arabinoside (Figure 6E). These findings collectively suggest that these TFs may play critical roles in regulating flavonoid biosynthesis in *L. chinensis* in response to saline–alkali stress.

## 3. Discussion

This study systematically demonstrated the saline–alkali tolerance mechanisms of *Leymus chinensis* variety Nongjing–4 through a joint analysis of its physiology, transcriptome, and metabolome under different intensities of saline–alkali stress. The results not only enriched the understanding of forage grass adaptation to environmental stresses but also provided a broader theoretical foundation for molecular breeding. The following will delve into physiological responses, molecular mechanisms, metabolic regulation, and flavonoids and their regulatory networks and conduct in–depth discussions in conjunction with relevant cutting–edge research.

### 3.1. Dual–Effect of Saline–Alkali Stress on the Growth of Leymus chinensis NQ–4

*Leymus chinensis* NQ–4 exhibited distinct growth and physiological traits under moderate (100 mM) and severe (150 mM carbonates) saline–alkali stress. Moderate saline–alkali stress did not inhibit but rather promoted plant growth by enhancing the activity of the antioxidant system. The contents of total flavonoids and phenolic acids in leaves significantly increased, while the activities of antioxidant enzymes were enhanced; therefore, the MDA content was reduced, indicating alleviated membrane oxidative damage. This is consistent with existing research [47] showing that moderate adversity promotes the activation of plant defense mechanisms.

In contrast, severe saline–alkali stress significantly hindered plant growth and disrupted the balance of antioxidant enzyme activities. This led to an increase in MDA content, suggesting exacerbated oxidative damage that exceeded the plant’s self–regulatory capacity. This phenomenon reflects the “dose–response relationship” of stress: moderate stress can induce positive adaptive responses, whereas severe stress results in systemic imbalance and cellular damage.

### 3.2. Transcriptome and Metabolome Analyses Reveal the Synergistic Adaptations in Metabolism and Signaling in L. chinensis NQ–4 Under Saline–Alkali Stress

Transcriptome analysis revealed significant changes in the gene expression profile of *L. chinensis* NQ–4 under varying saline–alkali stress conditions, with particular enrichment of genes involved in sugar, energy metabolism, aquaporin–mediated transport, and phytohormone signal transduction [48]. Moderate stress significantly activated genes associated with aquaporins, sugar transport, and energy metabolism, suggesting that plants maintain cellular osmotic potential and metabolic homeostasis by regulating water transport and energy supply, thereby enhancing adaptive capacity. Severe stress, in contrast, substantially enriched genes involved in redox regulation, hormone transduction signaling, and stress–responsive pathways. This indicates that as stress intensity increases, plants activate a broader defense network, including the remodulation of ABA, SA, and JA signaling, as well as the activation of ROS–responsive genes. These pathways are widely conserved in the saline–alkali stress responses in model plants such as *Arabidopsis thaliana* and rice, implying a synergistic relationship between hormone homeostasis and signaling and secondary metabolite biosynthesis [49], and that *L. chinensis* employs evolutionarily conserved molecular adaptation strategies.

Metabolomic analysis identified 1360 metabolites, covering diverse secondary metabolites including flavonoids, phenols, alkyls, and lipids. Saline–alkali stress significantly altered the accumulation patterns of flavonoids, phenols, and other substances: moderate stress induces a significant increase in flavonoid content, while high–intensity stress leads to decreased levels of specific flavonoids and phenols [11]. The observations in this study are consistent with previous research reports. Notably, the dynamic changes in flavonoid biosynthesis are accompanied by coordinated alterations in the expression of genes involved in their biosynthetic pathways [50]. Our findings further corroborate the central role of flavonoids in mediating plant responses to saline–alkali stress.

Specifically, the 298 flavonoids identified in the metabolome were categorized into 10 subcategories, including chalcones, flavonols, isoflavones, and anthocyanins. Previous studies have demonstrated that stress significantly upregulates the accumulation of flavonoids such as 6–hydroxykaempferol–3,6–O–diglucoside, quercetin–sinapyl–glucoside, and tamarixetin–3–O–glucoside, molecules that have been previously clarified to exert potent antioxidant and cytoprotective effects in other salt– and alkali–tolerant plant species [51]. Conversely, the contents of certain flavonoids, such as 3′–O–methyltricetin–7–O–glucoside, decreased under high–intensity stress, indicating that stress intensity dynamically regulates both the synthesis and degradation of flavonoid metabolites.

### 3.3. Flavonoid Synthesis Pathway and Its Transcriptional Regulatory Network Responsive to Saline–Alkali Stress

It is widely reported that the key synthase genes in the flavonoid biosynthesis pathway (e.g., *CHS*, *F3H*, *DFR*, and *ANS*) are strongly correlated with flavonoid metabolite accumulation, with their expression synchronously regulated under environmental stresses [52]. The MYB and bHLH families, along with their downstream MBW (MYB–bHLH–WD40) complexes, play pivotal roles in regulating flavonoid biosynthesis [53]. The regulatory mechanism of MBW complexes in flavonoid biosynthesis has been well characterized in diverse crops such as *Arabidopsis thaliana*, rice, alfalfa, and quinoa [54,55]. Our study identified 22 candidate transcription factor genes significantly correlated with flavonoid metabolites, including 16 bHLH and 6 MYB TF genes. For instance, several bHLH TF genes, such as *cluster–35938.0*, *cluster–39899.3*, and *cluster–45911.3*, were strongly correlated with downregulated flavonoids, while specific MYB TF genes, such as *cluster–25926.0*, correlated with upregulated flavonoids. This suggests that these TFs may be potential key regulators in mediating the saline–alkali stress response, thus serving as promising target genes in molecular breeding aimed at enhancing plant stress tolerance.

### 3.4. Stress Adaptation Capacity and Ecological Restoration Significance of Leymus chinensis

The saline–alkali tolerance of *L. chinensis* is attributed not only to its complex root system and ecological adaptability but also to its efficient molecular–level stress response mechanisms [56]. By activating energy metabolism, regulating sugar and water transport, enhancing antioxidant system functions, promoting flavonoid synthesis, and fine–tuning these processes via transcription factors, *L. chinensis* can achieve growth and metabolic homeostasis in saline–alkali soil. The “activation adaptation” mechanism under moderate stress provides a new idea for forage molecular breeding: by manipulating the flavonoid biosynthesis pathway and related transcription factors, it is feasible to develop new varieties with enhanced saline–alkali tolerance. The molecular mechanisms revealed in this study are of great significance for grassland ecological restoration, saline–alkali soil amelioration, and efficient utilization of forage resources. Furthermore, these findings offer a theoretical basis for the molecular breeding of saline–alkali–tolerant crops.

### 3.5. Research Limitations and Prospects

Although this study systematically elucidates the mechanism of saline–alkali tolerance in *L. chinensis*, several limitations still remain: Firstly, saline–alkali stress experiments were conducted under short–term (14–day) treatment conditions. Although this design captures the initial stress response, it fails to reflect the long–term adaptation strategies of *Leymus chinensis* in coping with persistent saline–alkali stress in its natural habitat, such as gradual morphological adjustments (such as root remodeling) or metabolic memory formation. This gap limits our understanding of the overall tolerance mechanisms of plants under different stress durations. Secondly, although the biosynthesis pathway of flavonoids has been identified as a key module for saline–alkali tolerance, the regulatory network controlling this pathway has not been fully resolved. The specific roles of individual TFs in this network have not been fully validated, such as their binding specificity to target gene promoters, their synergy or interaction with other regulatory proteins, and their temporal expression dynamics. If functional validation is not conducted through genetics, the causal relationship between these TFs and stress tolerance mediated by flavonoids remains speculative. Thirdly, the experiment was conducted under controlled environmental conditions, focusing only on saline–alkali stress and ignoring the interactions of other non–biological factors coexisting in natural ecosystems, such as temperature fluctuations, changes in light intensity, and humidity gradients. In fact, the interaction of multiple stresses (such as saline–alkali stress or other environmental factors) on *Leymus chinensis* may significantly alter its tolerance response. Neglecting these interactions limits the ecological relevance of current research results.

To address the limitations of this study, future research could pursue the following issues. Firstly, we should integrate multi–omics technologies (e.g., time–series transcriptomics, metabolomics, and epigenomics) with long–term stress treatment experiments to dissect the dynamic regulatory network underlying *L. chinensis*’s sustained saline–alkali tolerance. This approach would help identify late–stage adaptive genes and epigenetic modifications that drive long–term stress acclimation. Secondly, we should prioritize functional verification of key transcription factors and structural genes in the flavonoid biosynthesis pathway–using transgenic overexpression, gene knockout, and dual–luciferase reporter assays–to clarify their molecular functions and regulatory hierarchies. This would validate the causal relationships proposed in this study and refine the flavonoid–centered tolerance model. Thirdly, we should design multi–factor stress experiments that combine saline–alkali stress with other environmental variables (e.g., high temperature or drought) to explore the cross–stress response mechanisms of *L. chinensis*. These studies will enhance the ecological validity of the findings and provide insights into how the plant copes with complex field conditions. Additionally, the key functional genes identified in this study (e.g., flavonoid biosynthesis genes or stress–responsive TFs) could be applied to molecular breeding to develop *L. chinensis* varieties with enhanced and stable saline–alkali tolerance. This translation of basic research into practical applications will contribute to the restoration and sustainable management of saline–alkali grasslands.

## 4. Materials and Methods

### 4.1. Materials and Treatment

Nongjing–4 (NQ–4), a saline–alkali–tolerant *Leymus chinensis* variety, registered as a crop cultivar in Heilongjiang Province (Registration No. 2008008), was selected for comprehensive analysis. NQ–4 plants used in this study were derived from the same maternal plant via tissue culture and thus share an identical genetic background. The propagation of tissue–cultured seedlings is a time–consuming process since *Leymus chinensis* primarily achieves population expansion through creeping rhizomes that can sprout new shoots. Therefore, we used one–year–old plants for treatment. The one–year–old *Leymus chinensis* plants were treated with mixed carbonate solutions (Na_2_CO_3_:NaHCO_3_ = 1:9, mol–mol, corresponding to ~pH9.3) [11], at concentrations of 100 mM and 150 mM for 14 days. The experiment was conducted outdoors in Harbin, China (latitude: 45.71859°, longitude: 126.62573°), in August, with plants protected from rainfall. After treatment, the leaves were randomly collected for subsequent analysis.

### 4.2. Determination of Antioxidant Properties

The determination of antioxidant enzyme activities referred to Qian et al., 2023 [11]. Peroxidase (POD) activity: The guaiacol method is employed, utilizing POD to catalyze the decomposition of H_2_O_2_ and oxidize guaiacol. The rate of absorbance change at 470 nm is measured, and the enzyme activity is calculated. Superoxide dismutase (SOD) activity: The nitrogen blue tetrazolium (NBT) colorimetric method is employed, based on the ability of SOD to inhibit a reduction in NBT by superoxide anion radicals to form blue formazan. The absorbance is measured at 560 nm, and the enzyme activity is calculated. Catalase (CAT) activity: The H_2_O_2_ decomposition rate method was employed to measure the degradation rate of H_2_O_2_ at 240 nm, and the CAT activity was calculated accordingly. Determination of malondialdehyde (MDA) content: Using the thiobarbituric acid (TBA) method, MDA reacts with TBA to form a red compound, and the absorbance is measured at 532 nm to reflect the degree of lipid peroxidation and cell membrane damage.

### 4.3. Determination of Total Flavonoids and Phenolic Acids

The total flavonoid and phenolic contents were determined using the methods previously described with minor modification [57]. In total, 0.2 mg of leaf powder homogenized in 5 mL of 80% ethanol was extracted by ultrasonication at 70 °C. The absorbance of the water extract was measured at 510 nm. Rutin was used as a standard. The total phenolic content was determined using 0.2 mg of leaf powder by the Le method [58], with absorbance measured at 765 nm.

### 4.4. Transcriptome Sequencing and Analysis

Sample preparation: The *Leymus chinensis* leaves were collected randomly from the treatment groups (CK, SA100, SA150), quickly frozen in liquid nitrogen, and stored at −80 °C. RNA extraction and quality control: We utilized commercial plant RNA extraction kits to assess RNA integrity (e.g., using the Agilent 2100, Agilent Technologies, Santa Clara, CA, USA), concentration, and purity (with NanoDrop, Thermo Scientific, Waltham, MA, USA). Library construction and sequencing: We utilized the Illumina platform (such as NovaSeq 6000, Illumina, San Diego, CA, USA) to prepare a dual–ended cDNA library and obtain high–quality raw data (raw reads). Differential expression analysis: We used DESeq2 or edgeR to screen for differentially expressed genes (DEGs); the commonly used screening criteria are ∣log_2_FC∣ > 1 and FDR < 0.05. Enrichment analysis: The GO and KEGG databases were used for annotation and to classify the DAMs. GraphPad Prism 8 was used for graphic drawing. Duncan’s multiple range test was used to determine statistical significance with a *p*-value < 0.05; data are expressed as mean ± SD. The raw transcriptome reads were deposited in the NCBI Sequence Read Archive (SRA) database (PRJNA1359371).

### 4.5. Metabolomic Detection and Analysis

The metabolome preparation and detection method is similar to previous reports. Sample extraction: We took frozen leaves, 0.5 g per biological repeat, ground and sampled, and then extracted metabolites using methanol or a methanol–water mixed solution. After ultrasonic extraction, we centrifuged and collected the supernatant for detection. Metabolite identification used an ultra–performance liquid chromatography–tandem mass spectrometry (UPLC–ESI–MS/MS) platform (UPLC, SHIMADZU Nexera X2, Shimadzu, Kyoto, Japan). The parameters are basically the same as those of Qian et al., 2023 [57]. Identification of differentially accumulated metabolites (DAMs) and PCA (principal component analysis) were performed using the same method as a previous study [56]. Metabolite databases and standards were used for qualitative analysis, combined with multiple reaction monitoring (MRM) for quantification. Comprehensive screening of differentially accumulated metabolites (DAMs) was conducted using VIP values (PLS–DA analysis), fold change, and *p*-values. We conducted KEGG pathway enrichment analysis, with a focus on analyzing changes in secondary metabolites, flavonoids, phenols, and other substances.

### 4.6. Combined Omics Analysis

Common pathway analysis: We mapped DEGs and DAMs to KEGG pathways and screened for commonly enriched pathways. Correlation analysis: The Pearson correlation coefficient was used to calculate the expression correlation between genes and metabolites. A correlation is considered significant when the absolute value is greater than 0.8 and *p* < 0.05. Visualization: We drew nine–quadrant plots, correlation heatmaps, and network diagrams to reveal the synergistic regulatory relationships between genes and metabolites and to identify key regulatory factors.

## 5. Conclusions

**Figure 7:**
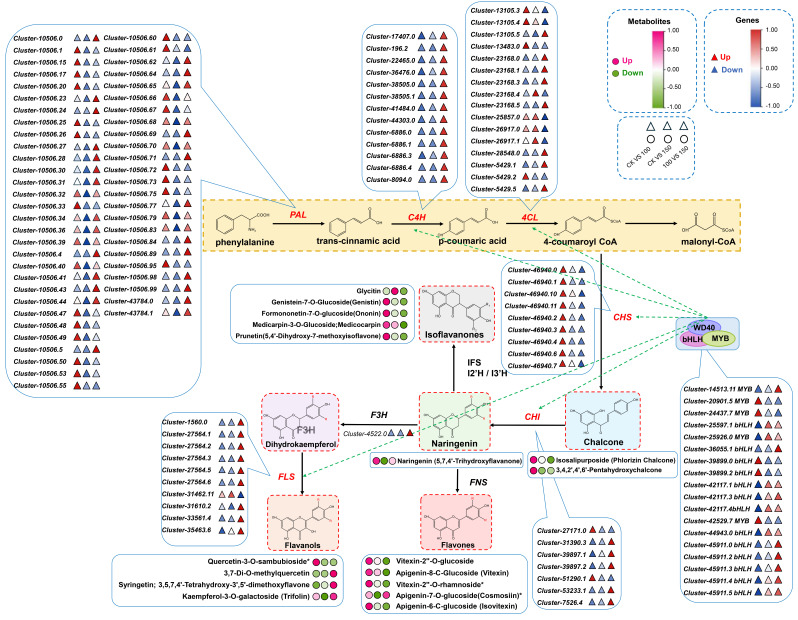
Schematic diagram of flavonoid biosynthesis and regulatory network response to saline–alkali stress in *Leymus chinensis*. Red and blue dots, gene expression levels. Green and pink dots, metabolite accumulation levels. Broken lines with arrows, targeted genes regulated by TFs in the MBW complex. * *p* < 0.05.

This study clarified the transcriptomic and metabolomic differences between moderate (SA100, growth–promoting) and severe saline–alkali stress (SA150, growth–inhibiting) groups to explain their contrasting growth phenotypes, innovatively revealing the “stress intensity–dependent growth response” pattern. At the transcriptomic level, moderate saline–alkali stress activated a “Growth–Homeostasis” strategy, with 9334 DEGs regulating osmotic potential and energy supply for growth support. In contrast, severe saline–alkali stress shifted to a “Survival–Defense” mode, where DEG numbers doubled to 19,096, prioritizing systemic stress alarms over growth support. Metabolomically, moderate stress upregulated total flavonoids and phenolic acids via flavone/flavonol biosynthesis, which, combined with the enhanced activities of antioxidant enzymes (SOD/POD/CAT) and reduced MDA content, provided growth maintenance. However, severe stress downregulated these key metabolites, accompanied by decreased CAT activity and increased oxidative damage, leading to upregulated MDA content. The core difference lies in the fact that moderate saline–alkali stress acts as an “Adaptive Activation” stimulus, promoting growth through the synergy of energy metabolism and antioxidant systems. In contrast, severe stress exceeds tolerance thresholds, causing “systemic imbalance” and ultimately growth retardation (Figure 7). Our findings provide key transcriptomic–metabolomic evidence for elucidating the dose–effect mechanism of plant responses to saline–alkali stress.

## Data Availability

The data presented in this study are openly available at the NCBISequence Read Archive (SRA) database (PRJNA1359371).

## Supplementary Materials

The following supporting information can be downloaded at https://www.mdpi.com/article/10.3390/plants14243852/s1: Table S1: The flavonoids detected in *L. chinensis* leaves in this study. Table S2: The primers used in this study. Figure S1: TIC overlap diagram of QC sample mass spectrometry.
