# Supplementary material for: Unraveling the Saline–Alkali–Tolerance Mystery of Leymus chinensis Nongjing–4: Insights from Integrated Transcriptome and Metabolome Analysis
Source: Plants (Basel). 2025 Dec 17;14(24):3852. doi: 10.3390/plants14243852 (PMC12736582; doi:10.3390/plants14243852)
Supplement: Supplementary file 1 [file plants-14-03852-s001.zip › Figure S1.pdf]

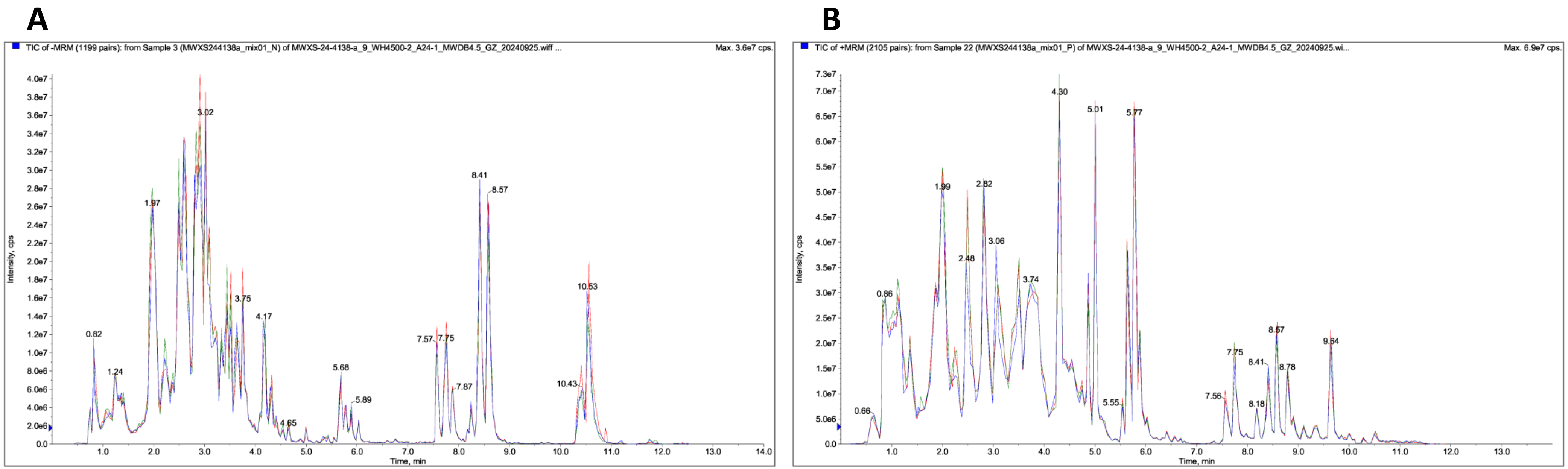

Fig. S1 TIC overlap diagram of QC sample mass spectrometry.

Superposition of total ion flow (TIC) diagram detected by mass spectrometry of QC samples. A. The negative ion mode. B. The positive ion mode.
